# Detection of Usutu, Sindbis, and Batai Viruses in Mosquitoes (Diptera: Culicidae) Collected in Germany, 2011–2016

**DOI:** 10.3390/v10070389

**Published:** 2018-07-23

**Authors:** Dorothee E. Scheuch, Mandy Schäfer, Martin Eiden, Eva C. Heym, Ute Ziegler, Doreen Walther, Jonas Schmidt-Chanasit, Markus Keller, Martin H. Groschup, Helge Kampen

**Affiliations:** 1 Friedrich-Loeffler-Institut, Federal Research Institute for Animal Health, Suedufer 10, 17493 Greifswald, Germany; ellen.scheuch@gmx.de (D.E.S.); mandy.schaefer@fli.de (M.S.); martin.eiden@fli.de (M.E.); ute.ziegler@fli.de (U.Z.); markus.keller@fli.de (M.K.); martin.groschup@fli.de (M.H.G.); 2 Leibniz-Centre for Agricultural Landscape Research, Eberswalder Str. 84, 15374 Muencheberg, Germany; eva.heym@zalf.de (E.C.H.); doreen.walther@zalf.de (D.W.); 3 Bernhard-Nocht-Institute for Tropical Medicine, Bernhard-Nocht-Str. 74, 20359 Hamburg, Germany; schmidt-chanasit@bnitm.de

**Keywords:** zoonoses, vector-borne, viruses, emerging diseases, One Health, globalization, mosquitoes, Sindbis virus, Usutu virus, Batai orthobunyavirus

## Abstract

Due to the emergence of non-endemic mosquito vectors and the recent outbreaks of mosquito-borne diseases, mosquito-borne pathogens are considered an increasing risk to public and animal health in Europe. To obtain a status quo regarding mosquito-borne viruses and their vectors in Germany, 97,648 mosquitoes collected from 2011 to 2016 throughout the country were screened for arboviruses. Mosquitoes were identified to species, pooled in groups of up to 50 individuals according to sampling location and date, and screened with different PCR assays for *Flavi-*, *Alpha-* and *Orthobunyavirus* RNA. Two pools tested positive for Usutu virus-RNA, two for Sindbis virus-RNA, and 24 for Batai virus-RNA. The pools consisted of *Culex pipiens* s.l., *Culex modestus*, *Culex torrentium*, *Culiseta* sp., *Aedes vexans*, *Anopheles daciae*, and *Anopheles messeae* mosquitoes and could be assigned to nine different collection sites, with seven of them located in northeastern Germany. Phylogenetic analyses of the viral RNA sequences showed relationships with strains of the viruses previously demonstrated in Germany. These findings confirm continuing mosquito-borne zoonotic arbovirus circulation even though only a rather small percentage of the screened samples tested positive. With respect to sampling sites and periods, virus circulation seems to be particularly intense in floodplains and after flooding events when mosquitoes develop in excessive numbers and where they have numerous avian hosts available to feed on.

## 1. Introduction

Due to globalization and climate warming, the number of cases and outbreaks of mosquito-borne diseases among humans and animals are likely to increase in Europe in the near future. After the recent emergence of both efficient invasive mosquito vectors, such as *Aedes aegypti* and *Aedes albopictus* [1], and putatively exotic mosquito-borne disease agents, such as dengue and chikungunya viruses [2], mosquito research has experienced a renaissance in many European countries following decades of neglect. In Germany, mosquitoes are on the agenda again as possible vectors of pathogens after several mosquito-borne viruses and dirofilarial worms were recently detected in and even isolated from mosquitoes collected in the country [3].

Nine mosquito-borne viruses have been shown to be endemic or temporarily circulate in Europe [2,4,5]: Usutu virus (USUV), Sindbis virus (SINV), Batai virus (BATV), Tahyna virus (TAHV), Inkoo virus (INKV), Lednice virus (LEDV), West Nile virus (WNV), chikungunya virus (CHIKV) and dengue virus (DENV). With the exception of LEDV, all of them have the potential to cause disease in humans or animals, or both. Most infections appear to pass unnoticed or present with transient mild febrile symptoms, commonly referred to as “summer flu” [5]. However, WNV, CHIKV, and DENV have also been associated with fatal disease [6,7,8]. Due to their occasional re-emergence, all nine viruses are apparently vectored by indigenous mosquito species, with the newly established invasive Asian tiger mosquito, *Ae. albopictus*, responsible for recent outbreaks of chikungunya and dengue [9].

About 50 mosquito species are known to occur in Germany, and many of these are thought to be vector-competent for various arboviruses and filarial nematodes [3]. As for viruses, only USUV BATV, SINV, and TAHV have so far been demonstrated to circulate. USUV, a mosquito-borne *Flavivirus*, has been repeatedly recorded in Germany since 2011, with several outbreaks among birds characterized by significant mortality [10,11]. In addition, a German blood donor tested positive for USUV infection in 2016 [12]. USUV is regarded primarily as an avian pathogen; however, human infections with involvement of the central nervous system have been observed [13,14]. BATV (genus *Orthobunyavirus*) infections of goats and SINV (genus *Alphavirus*) infections of birds and humans were also recently diagnosed in Germany [15,16,17]. Both viruses may cause an influenza-like illness in humans, accompanied by mild symptoms like headache, malaise, myalgia, arthralgia, rash, and fever [18,19].

TAHV infections were demonstrated in Germany in the late 1960s in both humans and mosquitoes [20,21], and in the early 1980s and mid-1990s again in mosquitoes [22,23]. Symptoms may include fever, pneumonia, arthralgia, myalgia, abdominal pain and, rarely, conjunctivitis, meningitis, and encephalitis [24,25].

Given the scarce data on the occurrence and ecology of mosquito vectors in light of a significant increase in outbreaks and cases of mosquito-borne disease in southern Europe, several mosquito and pathogen surveillance approaches were initiated recently in Germany. The purpose of these projects was to collect proper knowledge and reliable data about mosquito species distribution and vector competence in order to establish an early warning system for arboviral emergence in a One-Health approach. The present work summarizes the viral screening of mosquitoes collected over six years, from 2011 to 2016, in the framework of the German surveillance program.

## 2. Materials and Methods

### 2.1. Mosquito Collection and Identification

**Figure 1:**
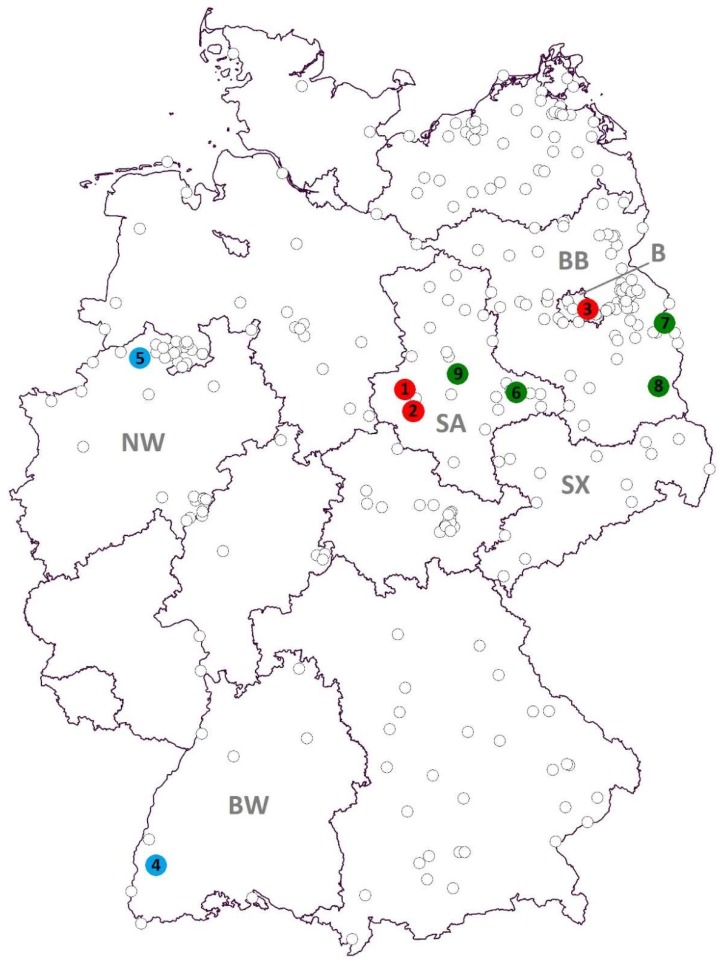
Mosquito sampling locations and viral RNA findings in Germany. Red = Sindbis virus (SINV), blue = Usutu virus (USUV), green = Batai virus (BATV), B = federal state of Berlin, BB = Brandenburg, BW = Baden-Wuerttemberg, NW = North Rhine-Westphalia, SA = Saxony-Anhalt, SX = Saxony.

From 2011 to 2016, mosquitoes were collected throughout Germany (Figure 1) using CO_2_-baited BG Sentinel (Biogents AG, Regensburg, Germany) and encephalitis virus surveillance (EVS) traps (Bioquip, Compton, CA, USA). Collection sites were randomly chosen in urban, rural, and natural environments. BG Sentinel traps were operated during the mosquito season from April to October for 24 h per week, while EVS traps were run occasionally overnight during the peak season (July to September) at locations with known high mosquito abundances. Additionally, collections were performed manually in animal shelters during the summer months and in mosquito hibernation sites during the winter months.

Mosquitoes were transferred to −20 °C or −80 °C as soon as possible after collection, i.e., immediately after emptying the traps. From manual collection activities in the field, the mosquitoes were brought home alive and only killed in the laboratory by deep-freezing. They were kept at freezing temperatures until DNA/RNA extraction, with morphological species identification being done on a chilling table.

With few exceptions, where collected mosquitoes were screened for viruses without identification below genus level, all mosquitoes were morphologically identified to species or species complex level using the determination keys by Schaffner et al. [26] and Becker et al. [27]. Individuals of the *Anopheles maculipennis* complex were specified by conventional PCR [28], while part of the individuals belonging to the *Culex pipiens* complex were subjected to a real-time PCR assay not only differentiating between *Cx. pipiens* and *Culex torrentium* but also between the two *Cx. pipiens* biotypes, *Cx. p.* biotype *pipiens* and *Cx. p.* biotype *molestus* [29]. Due to the large numbers of mosquitoes collected, however, the remaining *Cx. pipiens* complex specimens were not identified to species but further processed as “*Cx. pipiens* s.l.” (sensu lato).

### 2.2. DNA/RNA Extraction and Pathogen Screening

Mosquitoes that had to be identified genetically were individually subjected to DNA/RNA extraction. All other mosquito specimens, including those processed at the genus level, those morphologically determined, and the *Cx. pipiens* complex specimens, were pooled according to genus/species complex/species, sampling date, and sampling location for viral screening. Pools consisted of a maximum of 50 *Culex*, *Aedes*, or *Anopheles* specimens or 15 *Culiseta* or *Coquillettidia* specimens, depending on species size. Mosquito pools or single individuals were homogenized in the presence of three 3-mm steel beads and 750 µL (for mosquito pools) or 450 µL (for single mosquitoes) of serum-free minimum essential medium (MEM) in 2-mL Eppendorf tubes for 3 min at 30 Hz using a TissueLyser II (Qiagen, Hilden, Germany). Homogenates were centrifuged for 1 min at 13,000 rpm, and 200 µL of the supernatant were used for DNA/RNA extraction by the KingFisher Flex purification system (ThermoFisher, Darmstadt, Germany), using the NucleoMag VET Kit (Macherey & Nagel, Berlin, Germany) following the manufacturer’s instructions. The remainders of the homogenates were stored at −80 °C to be used for inoculation of culture cells in case of pathogen detection by PCR. DNA/RNA solutions were tested for viral RNA using several PCR assays: two SYBR Green-based quantitative real-time PanFlavi assays with different pairs of primers [30,31] coupled to melting curve analysis, and two multiplex quantitative real-time RT-PCR assays, with the first multiplex-PCR (Multiplex 1) targeting SINV, BATV, and CHIKV using primers published in [17,32,33], and the second (Multiplex 2) targeting TAHV and INKV, using primers described in [34]. Both assays were performed with the same protocol using the AgPath-ID One-Step RT-PCR Kit (ThermoFisher, Darmstadt, Germany). DNA/RNA solution volumes added to the reaction mixtures comprised 5 µL and originated from pool extractions or from mixtures of DNA/RNA solutions of up to 25 single specimens, containing 5 µL of each specimen. Both a no template control and a water control were used in each PCR assay as negative controls.

PCR amplicons of positive pools were cycle-sequenced in both directions using the BigDye Terminator v1.1 Cycle Sequencing Kit (Applied Biosystems, Foster City, CA, USA) and the PCR primers as sequencing primers. The obtained RNA fragments were purified using NucleoSEQ columns (Macherey & Nagel) and afterwards run on a 3130 Genetic Analyzer (Applied Biosystems).

For virus isolation, 100 µL of the supernatants of the mosquito homogenates were used to inoculate cell monolayers according to [16]. Two days post infection, pathogen-specific PCRs were conducted on the cell culture supernatants, with primers described in [16,35] for SINV and primers published in [36,37] for USUV and BATV.

### 2.3. Phylogenetic Analyses

For SINV, extracted RNA from mosquito homogenates was reverse transcribed using Maxima Reverse Transcriptase (ThermoFisher, Darmstadt, Germany) in the presence of random hexamer primers. Specific amplification was performed according to [35] to produce overlapping fragments, which were then processed by Sanger sequencing using MWG Eurofins Genomics (Ebersberg, Germany). Resulting sequence reads were checked for integrity and finally assembled to generate a full-length genome of the SINV isolate. The full-length genome sequence was used for phylogenetic analysis together with published full genome sequences (GenBank, Rockville, MD, USA).

USUV- and BATV-RNA was reverse transcribed and amplified using Superscript III (ThermoScientific) and corresponding USUV- or BATV-specific primers [36,37]. Sequencing and sequence analysis were performed as with SINV. Resulting partial sequences were subjected to phylogenetic analysis. USUV phylogenetic analyses were conducted based on 975 nucleotides of the viral envelope protein gene and 1828 nucleotides of the NS5 peptide gene, respectively [36]. BATV sequence analyses were performed using the 541 nucleotide viral S-segment [37]. In contrast to SINV, however, only partial sequences could be recovered for both USUV isolates. Unfortunately, continuous partial sequences, which are a prerequisite of a comprehensive phylogenetic analysis, could be generated only from disparate regions (envelope protein vs. NS5 protein). For this reason, two different phylogenetic trees were prepared and depicted. Neighbor-joining consensus trees were prepared, including full-genome sequences of virus isolates obtained from European samples (humans, birds, mosquitos) [16,35]. All trees were calculated using the neighbor-joining algorithm implemented in Geneious [38].

## 3. Results

In total, 97,648 mosquitoes in 4144 pools were subjected to viral screening. Twenty-nine pools (including single mosquitoes) consisting of seven mosquito taxa tested positive for arbovirus-RNA, three for SINV-, two for USUV- and 24 for BATV-RNA (Table 1). RT-qPCR Ct-values of mosquitoes considered positive were mostly around 30, but three pools/single mosquitoes showed particularly low Ct values of around 20 or below.

**Figure 2:**
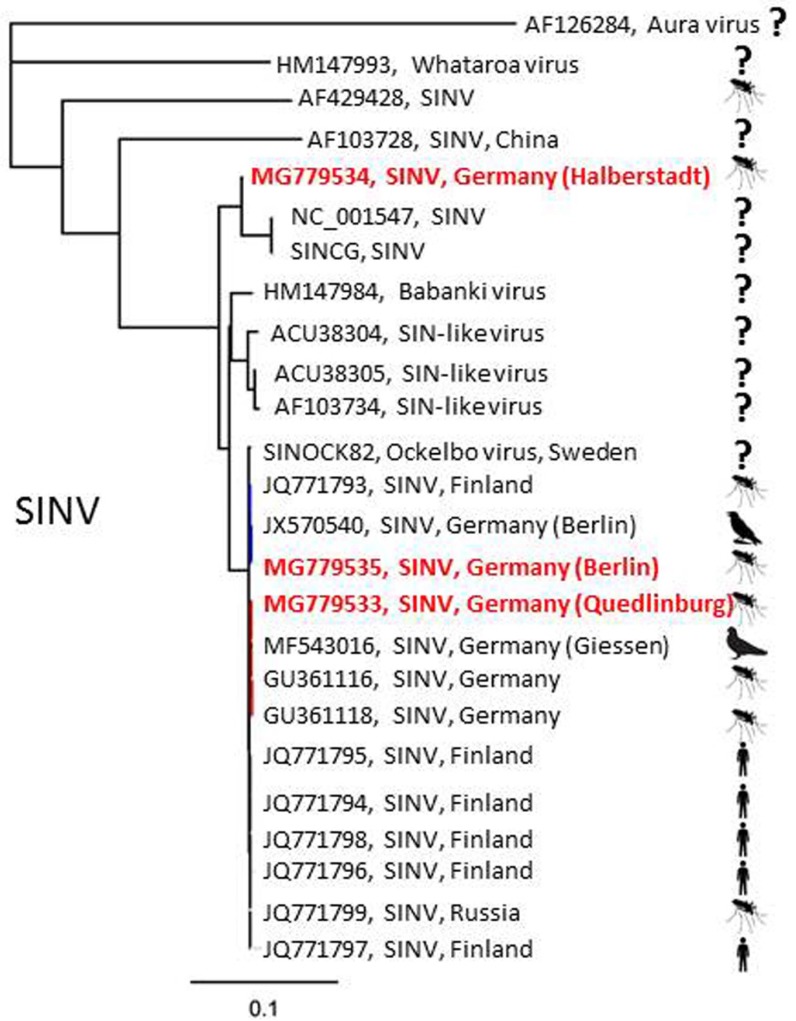
Phylogenetic tree based on full-genome sequences of SINV. Question mark = original source of the virus isolate is unknown to the authors. Red text marks samples of this study.

A pool of 30 *Cx. pipiens* s.l. mosquitoes, collected in July 2013 by a BG Sentinel trap in Halberstadt, in the German federal state of Saxony-Anhalt, tested positive for SINV-RNA. Two further SINV-RNA-positive samples, collected by an EVS trap, were found in 2015: one in July in Quedlinburg, Saxony-Anhalt, and the other in Berlin, with the latter consisting of 12 *Cx. pipiens* s.l. mosquitoes, showing a highly positive result (Ct = 20.50). The sample from Quedlinburg represented a single *Cx. torrentium* individual (Table 1). After applying the positive mosquito homogenates onto mammalian (Vero) and insect (C6/36) cells, a cytopathic effect was visible in the mammalian cells 48 hours post infection in the samples from Quedlinburg and Berlin. Specific SINV-PCR conducted two days post infection on the supernatants was positive for both cell lines and both samples. A phylogenetic analysis showed the virus isolate from Quedlinburg to be closely related to a SINV isolate found in Weinheim, Baden-Wuerttemberg, in 2010 [35] and the virus isolate of the Berlin sample to be closely related to a virus isolate obtained from a hooded crow that perished in Berlin in 2010 [16], but it was also more distantly related to the virus found in Halberstadt, which is only 14 km away from Quedlinburg (Figure 2).

**Figure 3:**
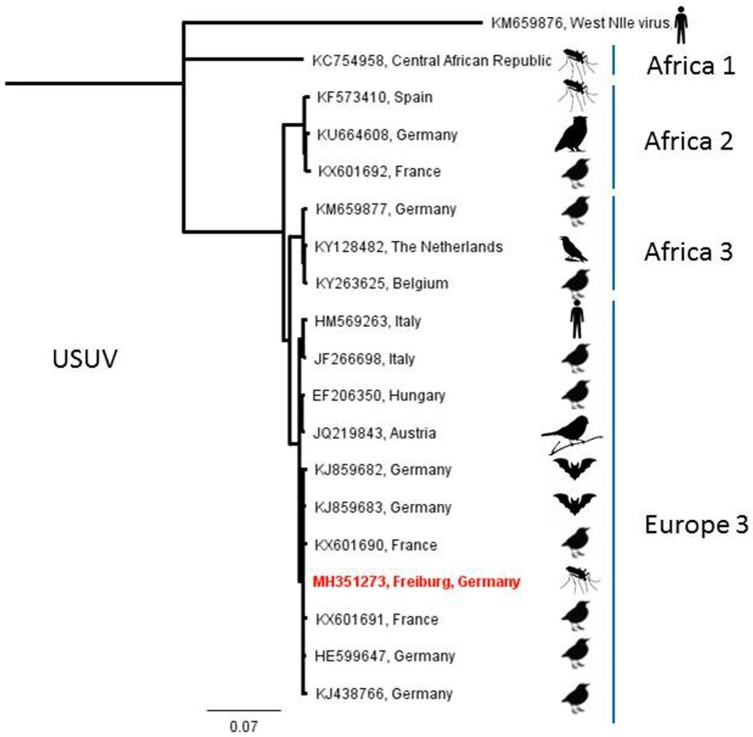
Phylogenetic tree of USUV sequences based on 975 nucleotides of the envelope protein. Red text marks sample of this study.

**Figure 4:**
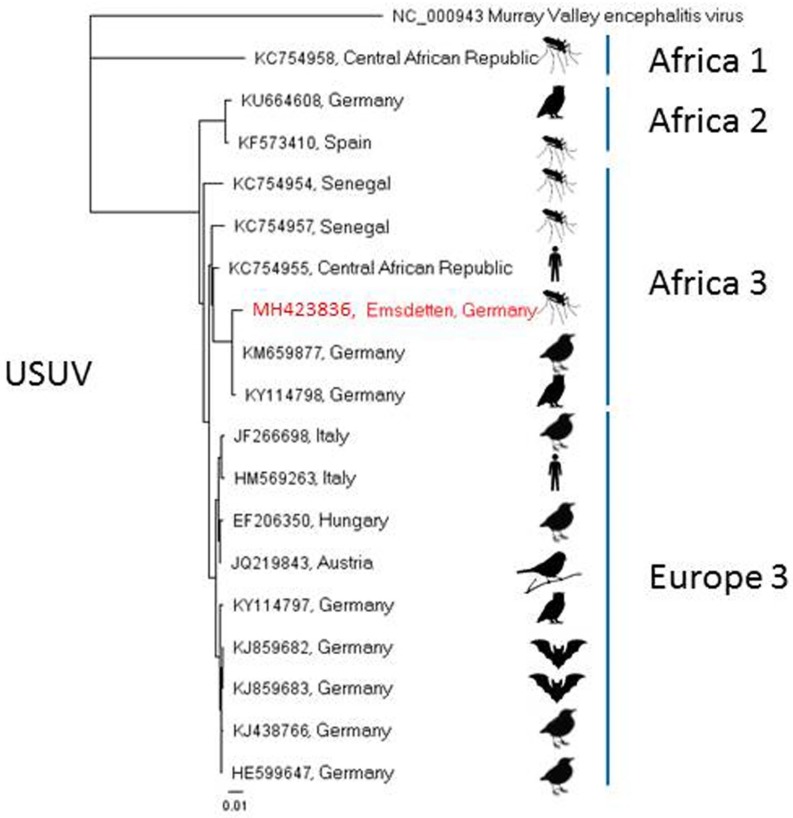
Phylogenetic tree of USUV sequences based on 1828 nucleotides of the NS5 protein. Red text marks sample of this study.

A pool of 32 *Cx. pipiens* s.l. mosquitoes trapped in September 2014 and a pool of 50 *Cx. pipiens* s.l. trapped in July 2016 by BG Sentinel traps in Freiburg, Baden-Wuerttemberg, and Emsdetten, North Rhine-Westphalia, respectively, showed positive signals in the PanFlavi-PCR (Table 1). Subsequent sequencing of the amplicons and comparison with GenBank data resulted in the detection of USUV-RNA. Attempts to amplify the virus in cell culture remained unsuccessful. Phylogenetic analyses of a recovered partial sequence classified the Freiburg sample to the USUV lineage “Europe 3” (Figure 3). According to phylogenetic analyses of the NS5 peptide gene sequence, the virus derived from Emsdetten clustered to the USUV lineage “Africa 3” (Figure 4).

The most frequently found viral RNA in the present investigation was that of BATV. One mosquito pool collected in 2012 as well as six mosquito pools and 17 individuals collected in 2013 at four different locations were positive for BATV-RNA (Table 1). The positive 2012 pool consisted of 15 hibernating *Culiseta* mosquitoes, not identified to species level, which had been collected by aspiration in January in the basement of an old deserted brewery building in Frankfurt/Oder, Brandenburg. Additionally, BATV-RNA was detected in a pool of 20 *Cx. pipiens* s.l. mosquitoes trapped in July 2013 by a BG Sentinel trap in Maust, Brandenburg. Further, three *Anopheles messeae* individuals, one *Anopheles daciae* specimen, and a pool of 25 *Cx. pipiens* s.l., collected in July and August 2013 by the same BG Sentinel trap in Seegrehna, Saxony-Anhalt, were positive for BATV-RNA. Finally, four mosquito pools and 13 single individuals from an EVS trap operated in August 2013 in Schönebeck/Elbe, Saxony-Anhalt, were found to be BATV-RNA-positive. Two of the Schönebeck/Elbe pools consisted of 24 and 33 *Aedes vexans*, respectively, the other two of 44 *Cx. modestus* and 27 *Cx. pipiens* s.l., while of the single mosquitoes, seven were *An. daciae* and six were *An. messeae*. Of the *An. daciae*, two specimens produced particularly low Ct values (16.13, 21.83).

**Figure 5:**
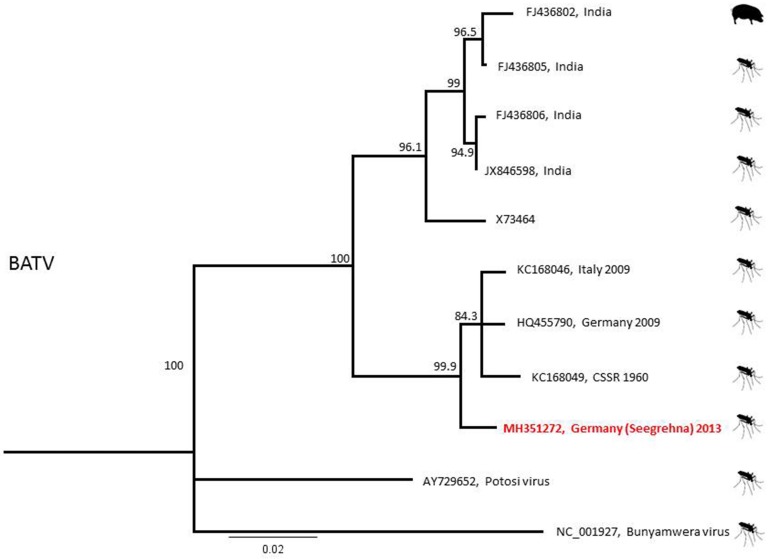
Phylogenetic tree of BATV based on the 541 nucleotide viral S-segment. Red text marks samples of this study.

BATV phylogeny showed the clustering of a Seegrehna sample with European BATV sequences from Italy and Germany (both 2009) and from the former CSSR (1960; Figure 5). Unfortunately, all attempts to cultivate the BATV-positive homogenate samples in different cell lines remained unsuccessful.

## 4. Discussion

In a six-year investigation (2011–2016), three zoonotic arboviruses were found circulating in the German mosquito fauna, though none of them for the first time. USUV RNA from a sample from Freiburg, Baden-Wuerttemberg (Figure 1), could be assigned to the “Europe 3” lineage, which has been shown to be endemic in southwestern Germany since 2011 [39]. Another USUV-RNA-positive sample from Emsdetten, North Rhine-Westphalia, belonged to the “Africa 3” strain and, thus, fits into the geographical context, as this strain has been reported from northwestern Germany, the Netherlands, Belgium, and France [40]. Cadar et al. [40] discuss the presence of these two USUV lineages in the Netherlands and France as the result of an introduction event from Germany. Also occurring in Germany are the “Africa 2” strain, which seems to be restricted to Berlin, and the “Africa 3-like” strain, which has been reported from Leipzig, Saxony [11,40]. While in this study USUV-RNA was only demonstrated in two mosquito pools from central western and southwestern Germany, USUV presently seems to be the most prevalent mosquito-borne virus in Germany, according to the data available [10,40,41]. The two positive sites are located within present hot spots of distribution which correlate with areas predicted suitable for USUV distribution [41].

The three SINV-RNA findings of the present study are all of the Palearctic/Ethiopian strain, which, in addition to the Oriental/Australian strain, is one of two known SINV strains [42,43]. Prior to this study, SINV circulation had sporadically been documented by virus detection in mosquitoes (2009) and serologically in asymptomatic humans (2010/2011) in the northern Upper Rhine Valley, southwestern Germany [17,35]. Also in 2009, BATV had been isolated from mosquitoes collected in the same region [32]. In the present study, BATV-RNA was the most frequently detected viral RNA, and a correlating antibody seroprevalence was eventually found in ruminants in the same regions of northwestern Germany [15]. Thus, BATV appears to be not only more prevalent but also more widely distributed in Germany than SINV.

All positive mosquito species found during the investigation are ubiquitous in Germany. *Aedes vexans* is a known inhabitant of floodplain forests and tends to develop massively after floodwater events like the Elbe flood in 2013, when the positive samples were collected. Members of the widely distributed *An. maculipennis* complex, which is associated with animal husbandry, were found to be carrying BATV-RNA (*An. messeae* and *An. daciae*). Their development in 2013 might also have benefitted from the Elbe flood. Out of 28 BATV-RNA-positive pools, six each consisted of *Cx. pipiens* complex and *An. maculipennis* complex mosquitoes. The same species complexes were previously found by Jöst et al. [32,35,44] as carriers of SINV, BATV, and USUV, respectively, and are thought to be vectors of several other zoonotic pathogens [45]. Both these complexes are considered widespread and highly abundant in Germany, with members feeding both on animals and humans, and may therefore pose a potential risk to public and animal health. Among others, the *Cx. pipiens* complex played a major role in the WNV epidemic in the USA starting in 1999 in New York [46], and indigenous populations have been shown to be susceptible to WNV infection in the laboratory [47].

Two *Ae. vexans* pools have been found BATV-RNA-positive in the present study. *Aedes vexans* has been linked to numerous pathogens [45] and has experimentally been shown to be able to transmit Rift Valley fever and Zika viruses [48,49].

The investigation of the indigenous mosquito fauna and its screening for pathogens has long been neglected in Germany, and data from the last few decades are scarce. Therefore, the results of this study cannot be put in a temporal context, and it is impossible to tell if the circulation of potentially zoonotic viruses in the German mosquito fauna has increased or spread over time. Fortunately, only viruses of minor pathogenicity have been detected so far.

As changes in the German mosquito fauna have recently become obvious, with increasing numbers of invasive species with known vector potential for arboviruses having been discovered [50,51,52], mosquito research, including both surveillance and vector competence studies, has returned to the scientific agenda. The present study underlines the importance of indigenous mosquitoes as possible vectors.

The Elbe flood is a possible explanation for the relatively high number of pathogen findings in 2013, as flood events favor mosquito mass development while high numbers of mosquitoes facilitate intensified virus circulation. A flooding setting is therefore likely to increase the chances to find infected mosquito specimens provided viruses and reservoir/amplifying vertebrate hosts are present. Epidemiologically, flooding events and similar extreme weather situations producing mosquito habitats, which are likely to increase in the future [53], might increase the risk of mosquito-borne disease agent transmission.

Except for one mosquito pool, all positive samples were collected in late summer, namely from July to September. These months are commonly considered the period of highest virus circulation in Europe, as the natural transmission cycles involving birds, insects and pathogens, which comes to a standstill in the vector-free period in winter and starts with the blood-feeding activity of the first mosquito generation in early spring each year, needs some time to keep going each season. Late summer also provides the most favorable climatic conditions in terms of temperature for mosquito and pathogen multiplication and propagation. In summer, floodplains also offer ideal conditions for huge numbers of resting and breeding birds which are appropriate blood hosts for the mosquito species found positive and reservoir hosts for several viruses [27]. Thus, the likelihood for humans and animals to become infected with a mosquito-borne virus increases with the proceeding summer. The single positive mosquito pool not found during late summer was collected in a hibernation shelter in January, underlining the role of overwintering mosquitoes as pathogen reservoirs c.f. [54].

Given the increasing international trade and travel, the occurrence of mosquito-borne arboviruses in Germany is likely to grow. In the future, the viruses demonstrated here might be supplemented by more pathogenic imported ones such as dengue, chikungunya, or Zika viruses. A continuation of mosquito surveillance and the screening of collected mosquitoes for pathogens as well as antibody screenings of sentinel animal hosts is therefore strongly recommended in the years to come in order to provide a mosquito-borne disease early warning system and to design adequate contingency plans. The One Health concept aiming at the creation of both a healthy environment and healthy human and animal populations reflects these needs.

## Figures and Tables

**Table 1:** Mosquitoes tested positive for zoonotic arbovirus-RNA. The abbreviation s.l. (= sensu lato) marks species complexes. Collection site numbers in parentheses refer to locations in Figure 1.

Detected Virus	Collection Site (Number in Figure 1)	Collection Date	Mosquito Species	Number of Pools (Pool Size)
SINV	Halberstadt (1)	28 July 2013	*Culex pipiens* s.l.	1 (30)
	Quedlinburg (2)	29 July 2015	*Culex torrentium*	1 (1)
	Berlin (3)	25 July 2016	*Culex pipiens* s.l.	1 (12)
USUV	Freiburg (4)	4 September 2014	*Culex pipiens* s.l.	1 (32)
	Emsdetten (5)	15 July 2016	*Culex pipiens* s.l.	1 (50)
BATV	Seegrehna (6)	4 July 2013	*Anopheles messeae*	1 (1)
		17 July 2013	*Anopheles daciae*	1 (1)
			*Anopheles messeae*	1 (1)
		21 August 2013	*Anopheles messeae*	1 (1)
			*Culex pipiens* s.l.	1 (25)
	Frankfurt/Oder (7)	13 January 2012	*Culiseta* sp.	1 (15)
	Maust (8)	28 July 2013	*Culex pipiens* s.l.	1 (20)
	Schönebeck/Elbe (9)	1 August 2013	*Anopheles messeae*	6 (1)
			*Anopheles daciae*	7 (1)
			*Aedes vexans*	1 (24)
			*Aedes vexans*	1 (33)
			*Culex modestus*	1 (44)
			*Culex pipiens* s.l.	1 (27)
